# The relationship between ventilatory ratio (VR) and 28-day hospital mortality by restricted cubic splines (RCS) in 14,328 mechanically ventilated ICU patients

**DOI:** 10.1186/s12890-022-02019-6

**Published:** 2022-06-13

**Authors:** Yingying Yang, Yi Chi, Siyi Yuan, Qing Zhang, Longxiang Su, Yun Long, Huaiwu He

**Affiliations:** Department of Critical Care Medicine, Peking Union Medical College Hospital, Peking Union Medical College, Chinese Academy of Medical Science, Beijing, 100730 China

**Keywords:** Ventilatory ratio, ICU mortality, Restricted cubic spline

## Abstract

**Background:**

Previous studies found that high levels of ventilatory ratio (VR) were associated with a poor prognosis due to worse ventilatory efficiency in acute respiratory distress syndrome patients. However, relatively few large studies have assessed the association between VR and intensive care unit (ICU) mortality in the general adult ventilated population.

**Methods:**

The present study is a retrospective cohort study. Patients mechanically ventilated for more than 12 h were included. VR was calculated based on a previously reported formula. Restricted cubic spline models were used to fit the relationship between VR and mortality risks.

**Results:**

A total of 14,328 mechanically ventilated ICU patients were included in the study, of which 1311 died within 28 days. The results of the study are as follows: (1) In the general adult ventilated population, VR was positively associated with 28-day mortality when VR ≥ 1.3 (increase of 0.1 per VR; HR 1.05, *p* < 0.001). The same tendency was also observed in the populations of severe hypoxemia with a PaO_2_/FiO_2_ (P/F) ratio < 200 mmHg. (2) However, in the population with a P/F ratio ≥ 200, a J-shaped dose–response association between VR and the risk of mortality was observed, with the risk of death positively associated with VR when VR ≥ 0.9 (10% increase in HR for every 0.1 increase in VR, *p* = 0.000) but negatively associated with VR when VR < 0.9 (10% decrease in HR for every 0.1 increase in VR, *p* = 0.034). In the population of P/F ratio ≥ 200 with VR less than 0.9, compared to the survival group, the nonsurvival group had a lower level PCO_2_ (33 mmHg [29.1, 37.9] vs. 34.4 mmHg [30.6, 38.5]), rather than a significant level of measured minute ventilation or P/F ratio.

**Conclusions:**

VR was positively associated with the risk of death in the general ICU population; however, VR was inversely associated with 28-day mortality in the population with a P/F ratio ≥ 200 and low VR . Further research should investigate this relationship, and VR should be interpreted with caution in clinical practice.

## Background

Gas exchange measurements are essential for understanding pulmonary physiology in critically ill patients on mechanical ventilation. Studies have found that impaired gas exchange is associated with the severity of pulmonary pathological diseases and could predict poor outcomes [1, 2]. The arterial partial pressure of oxygen/fraction of inspired oxygen (PaO_2_/FiO_2_) ratio is one of the most commonly used indicators of gas exchange, but it is a poor predictor of ICU mortality [3, 4]. The physiological dead-space fraction (VD/VT), which reflects CO_2_ clearance, is another important index of gas exchange. VD/VT has been shown to be a robust predictor of morbidity and extubation failure in the ICU [5, 6]. However, the measurements of VD/VT at the bedside are often inconvenient and expensive, which limits their applications in developing countries. The ventilatory ratio (VR), which was calculated as the ratio between the product of actually measured minute ventilation and PCO_2_ and the product of the predicted minute ventilation and PCO_2_ (Formula I), was first proposed by Sinha et al. [7]. In this formula, predicted minute ventilation was represented by [100 ml * predicted body weight (kg)]/min, and predicted PaCO_2_ was taken as 5 kPa. Predicted body weight (PBW, kg) was calculated using the ARDSnet PBW calculator. (Formula II) VR has been proven to be positively associated with dead space, and the correlations were stronger in patients under mechanical ventilation. Moreover, VR was confirmed as an independent predictor of ICU mortality in ARDS patients. Therefore, VR has emerged in recent years as a clinically useful tool for assessing impaired ventilation [7]. However, previous studies normally used the mean value or median level of VR as a cutoff point, and the cutoff points for predicting ICU mortality were not consistent across different studies. One study used the mean value of VR (VR = 1.4, N = 154) [8] and another study used the 60^th^ percentile of VR (VR = 2.1, N 1307) [9], and a recent study used the median level of VR (VR = 2.0, N = 520) [10] as a cutoff point to predict the relationship between VR and mortality. These studies led to the conclusion that a higher VR was associated with a higher mortality rate in ARDS patients [8, 10]. As VR is a continuous variable and the relationship between VR and all-cause mortality among critically ill patients was nonlinear, the choice of a cutoff point will affect the shapes of the dose–response relationships between VR and mortality. RCS models have been proven to be a good tool to fit the nonlinear association between continuous variables and outcomes. Studies have confirmed that RCS together with the Cox proportional hazards regression model is able to visualize the shapes of dose–response associations between a continuous variable and outcome risks [11, 12]. Therefore, the present study intends to explore the shape of the causal relation between VR and mortality in a mechanically ventilated ICU population using RCS regression models and determine the cut point of VR in a heterogeneous clinical population.

1 $$VR = \frac{{ \dot{V}E_{measured} \times PaCO2_{measured} }}{{\dot{V}E_{predicted} \times PaCO2_{predicted} }}$$

2 $$VR = \frac{{ \dot{V}E_{measured} \,\left( {\text{ml/min}} \right) \times PaCO2\,\left( {{\text{kPa}}} \right)}}{{100\,\left( {\text{ml/min}} \right) \times PBW\left( {{\text{kg}}} \right) \times 5\left( {{\text{kPa}}} \right)}}$$

## Methods

### Patients

**Fig. 1:**
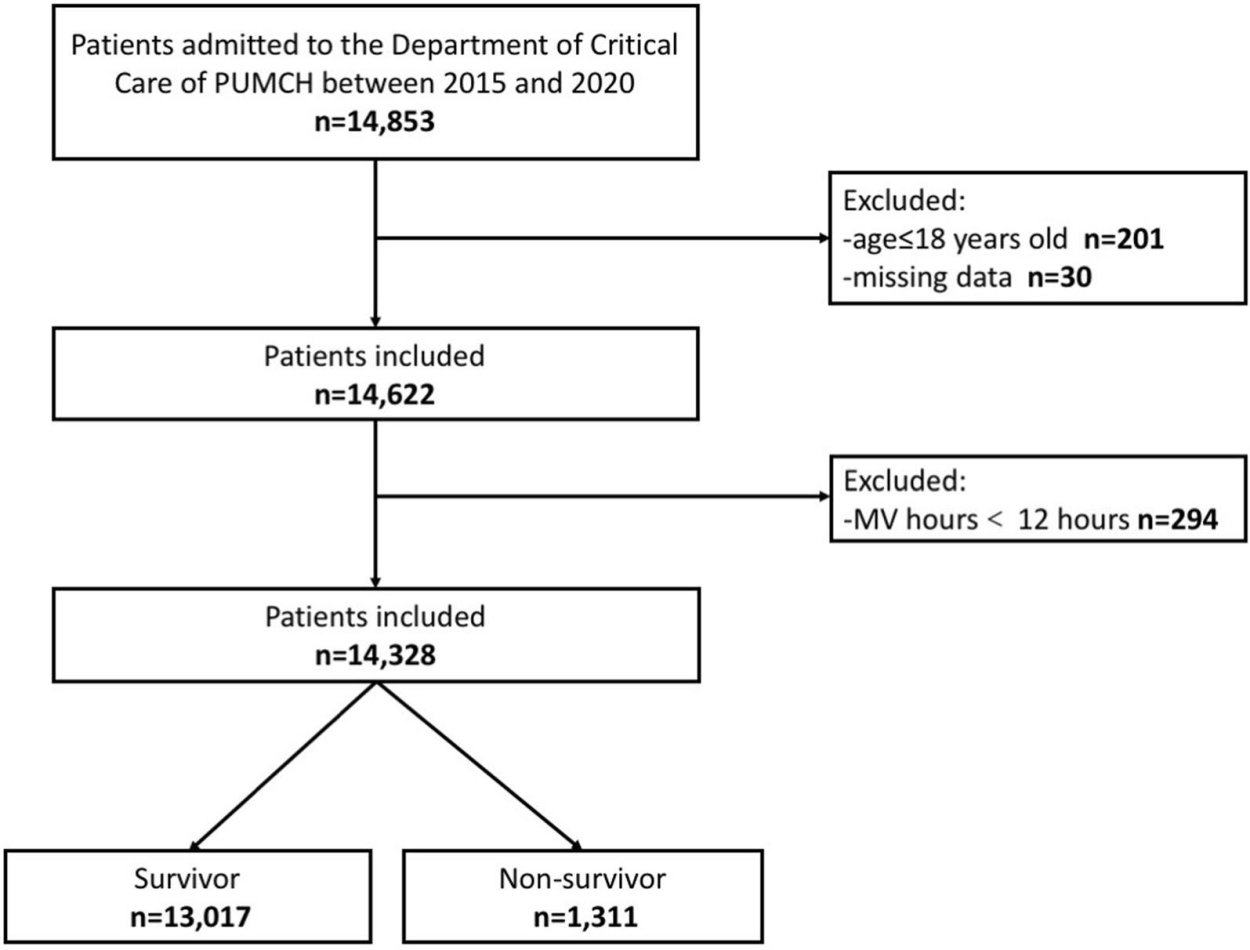
Flow chart for patient selection and inclusion and exclusion criteria in this study

This study was a retrospective study, and patients admitted to the Department of Critical Care of Peking Union Medical College Hospital (PUMCH) between 2015 and 2020 were enrolled. The inclusion criteria for the subjects were as follows: (1) Ages older than 18 years old. (2) The duration of mechanical ventilation was greater than 12 h (Fig. 1). Our center did not admit COVID-19 patients, so COVID-19 cases were not included in this study.

## Data collection

The baseline demographic and clinical characteristics were collected from electronic medical records and intensive care patient data management systems. The following demographic data were included: age, sex, surgery, Acute Physiology and Chronic Health Evaluation score (APACHE II), total days of mechanical ventilation, duration of hospital and ICU days, and survival outcome. The following intensive care system data were included: mean arterial pressure (MAP), heart rate (HR), central venous pressure (CVP), venous–arterial PCO_2_ (Pv–aCO_2_), central venous oxygenation (ScvO_2_), lactate (Lac), perfusion index (PI), respiratory rate (RR), expiratory tidal volume (Vte), minute ventilation, peak inspiratory airway pressure (Ppeak), mean airway pressure (Pmean), PCO_2_, partial pressure of oxygen/fraction of inspired oxygen ratio (PaO_2_/FiO_2_ ratio), and Sequential Organ Failure Assessment (SOFA).

### Measurement

In our study, the measurement of hemodynamic indices and respiratory mechanics measurements were registered concurrently with the blood draw for gas analysis (ABL300, Radiometer; Copenhagen, Denmark). In order to reduce the impact of the effect that all variables changed with time, the average values of the relevant variables 12 to 18 h after mechanical ventilation initiation were used in the final analysis. VR was calculated using the average values with the formula II.

### Statistical analysis

Continuous variables were tested for normality of the distribution with the Kolmogorov–Smirnov test. Normally distributed data were compared using the t test, whereas the Mann–Whitney U test was used to compare nonnormally distributed data. Restricted cubic spline (RCS) models were used to evaluate potential nonlinear associations between the hazard ratio (HR) and VR. Survival data were plotted as Kaplan–Meier (KM) curves with median survival and Cox proportional hazards regression (Cox regression). Univariate multivariate Cox regression analyses with RCS were performed using R version 4.0.2.

## Results

### Baseline demographic and clinical characteristics

A total of 14,328 patients were included in this study, of whom 1311 died during the ICU stay. A total of 52.7% of patients were males, and 78.7% of patients were treated surgically prior to admission to the ICU. The basic characteristics of the survival and non-survival groups are summarized in Table 1. In summary, the median length of ICU stay (9.0 vs. 4.0 days) and the duration of mechanical ventilation (186.0 vs. 65.0 h) in the nonsurvival group were significantly longer than those in the survival group. Regarding hemodynamics, the nonsurvival group displayed a higher HR, CVP, and PA-aCO_2_ as well as lower levels of MAP and PI than the survival group. In terms of baseline respiratory parameters, Ppeak, pmean, and VR were significantly higher, and the P/F ratio was significantly lower in the nonsurvival group than in the survival group.

**Table 1:** Baseline clinical characteristics and demographics of patients

Items	Total (N = 14,328)	Survivor (N = 13,017)	Non-survivor (N = 1311)	*p* value
Age, median (IQR), years	61.0 (49.0, 71.0)	59.0 (47.0, 69.0)	63.0 (53.0,73.0)	0.001
Male, No. (%)	7554 (52.7)	6747 (51.8)	807 (61.6)	0.049
Surgery, No. (%)	11,273 (78.7)	10,656 (81.9)	617 (47.1)	0.000
ICU days, median (IQR), day	2.0 (2.0, 4.0)	4.0 (2.0, 7.0)	9.0 (3.0, 17.0)	0.000
Ventilator hours, median (IQR), h	23.0 (19.0, 63.0)	65.0 (24.0, 141.0)	186.0 (57.5, 367.0)	0.000
SH, No. (%)	1398 (9.8)	1028 (7.9)	370 (28.2)	0.000
HR, median (IQR), bpm	85.5 (74.4, 97.3)	93.5 (82.9, 104.7)	101.5 (87.0, 114.3)	0.000
MAP, median (IQR), mmHg	90.9 (84.4, 97.9)	88.7 (83.4,94.2)	83.5 (77.9, 90.1)	0.000
CVP, median (IQR), mmHg	8.0 (6.5, 9.5)	8.3 (7.0, 9.7)	9.6 (7.7, 11.7)	0.000
PA-aCO2, median (IQR), mmHg	5.5 (3.6, 7.4)	5.6 (3.6, 7.5)	5.0 (3.1, 7.0)	0.027
ScvO2, median (IQR), %	75.1 (68.4, 81.4)	75.4 (68.7, 81.6)	73.6 (65.6, 80.6)	0.000
Lac, median (IQR), mmol/l	1.7 (1.1, 3.0)	2.7 (1.5, 5.0)	2.4(1.5, 5.5)	0.000
PI, median (IQR)	2.1 (1.3, 3.3)	1.5 (0.9, 2.4)	0.9 (0.5,1.7)	0.000
RR, median (IQR), bpm	15.4 (14.2, 16.9)	15.3 (14.1, 16.7)	18.0 (15.4, 21.2)	0.000
Vte, median (IQR), ml	407.0 (371.3, 449.0)	408.3 (373.7, 449.1)	405.6 (366.3, 458.3)	0.672
Ppeak, median (IQR), mmHg	17.3 (15.3, 19.6)	17.2 (15.3, 19.4)	20.0 (17.0, 23.8)	0.000
Pmean, median (IQR), mmHg	8.4 (7.8, 9.3)	8.4 (7.8, 9.1)	10.1 (8.7, 12.6)	0.000
P/F ratio, median (IQR), mmHg	346.8 (258.3, 437.5)	347.5 (260.9, 442.5)	237.5 (162.4, 347.1)	0.000
PCO2, median (IQR), mmHg	37.8 (33.9, 41.8)	37.9 (34.0,41.8)	37.3 (32.5, 42.9)	0.000
VR, median (IQR)	1.1 (0.9, 1.3)	1.1 (0.9, 1.2)	1.2 (1.0, 1.5)	0.001
SOFA, median (IQR)	4.0 (1.0, 7.0)	4.0 (2.0, 7.0)	9.0 (4.0, 13.0)	0.000
APACHEII, median (IQR)	13.0 (10.0, 17.0)	13.0 (10.0, 17.0)	23.0 (17.0, 29.0)	0.000

SH, severe hypoxemia; ScvO2, central venous oxygen saturation; Lac, lactate; PI, perfusion index; RR, respiratory rate; Vte, expiratory tidal volume; Vte, inspiratory tidal volume; Ppeak: peak inspiratory airway pressure;Pmean,mean airway pressure; P/F Ratio,PaO2 (partial pressure of oxygen) /FiO2 (the fraction of inspired oxygen) ratio; APACHE, Acute Physiology and Chronic Health Evaluation; SOFA, Sequential Organ Failure Assessment

### The relationship between VR and ICU mortality in the total population

**Fig. 2:**
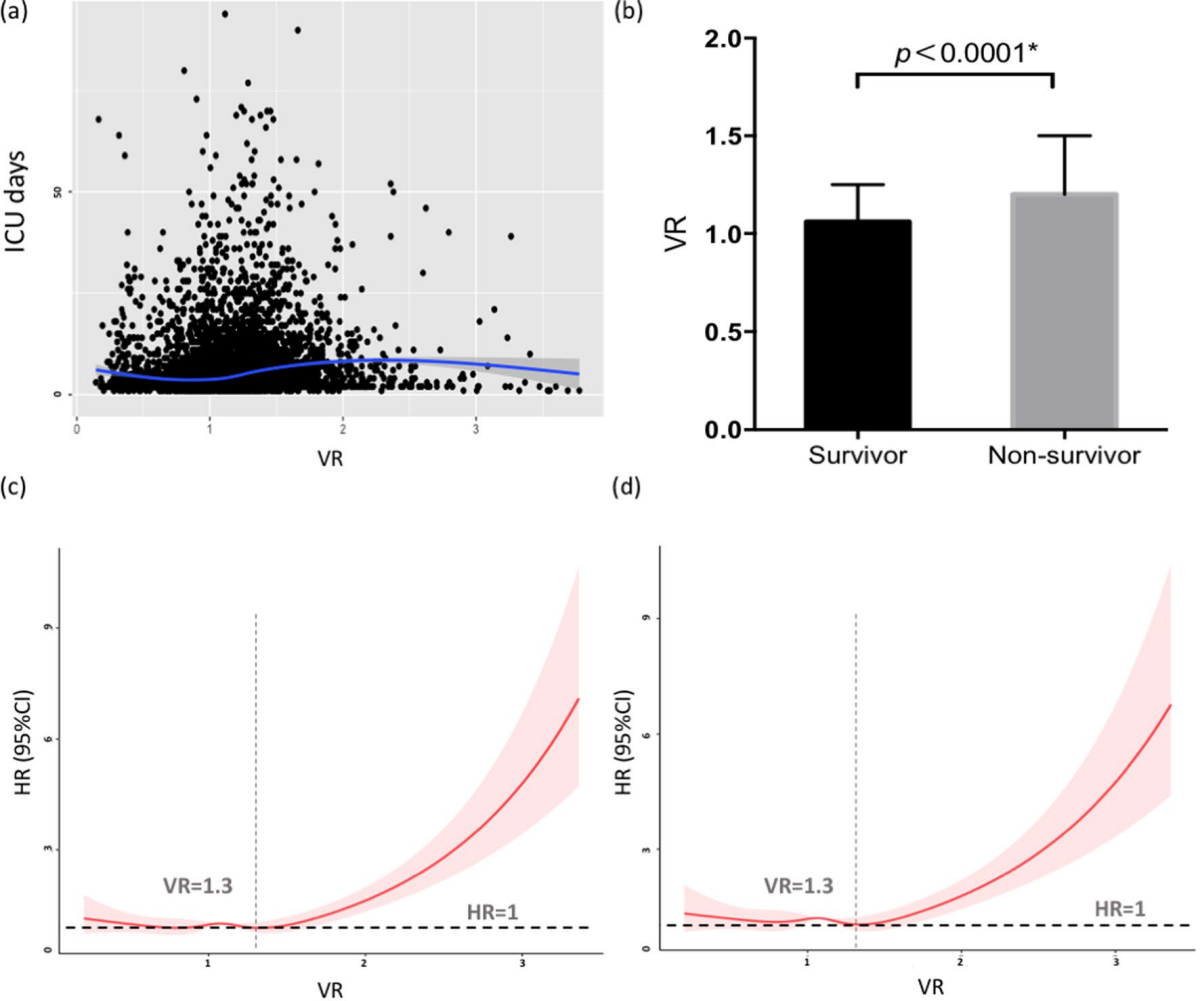
Association between VR and mortality in the total ICU population. **a** Scatter plot showing the relationship between VR and ICU days. The blue line with the gray area represents the 95% confidence intervals for the fitted nonlinear trend (*p* < 0.001). **b** VR in survivor and nonsurvivor group. The VR levels were significantly higher in the nonsurvivor group than in the survivor group by the Mann–Whitney nonparametric test (*p* < 0.0001). **c** The curve of HR versus VR using univariable Cox regression with restricted cubic splines. A Cox model with restricted cubic splines identified the lowest mortality risk when VR was 1.3. There were significant positive correlations between VR and HR for VR larger than 1.3 (increase of 0.1 per VR; HR 1.05, 95% CI 1.04–1.07). **d** The curve of HR versus VR using multivariable Cox regression with restricted cubic splines. The curve indicated a nadir of 1.3 even after adjusting for PEEP and the P/F ratio, and a highly positive correlation between VR and HR was also observed when VR was greater than 1.3 (increase of 0.1 per VR; HR 1.11, 95% CI 1.08–1.14). Cubic spline curves are shown as a solid line, with the shaded area representing the 95% confidence intervals

A scatter plot (Fig. 2a) was used to display the relationship between VR and ICU days, and the nonlinear test indicated that the relationship was significantly nonlinear (*p* < 0.001). Compared with the alive group, the non-survival group had significantly elevated VR (1.20 vs. 1.06, *p* < 0.0001) (Fig. 2b). To evaluate the impact of VR on nonlinear trends of death risk, RCS was incorporated into the Cox models. The results of univariable Cox regression with RCS showed the HR was approximately close to one until 1.3 of VR and a highly significant positive correlation between VR and HR was presented from the onset of 1.3 of VR (HR = 1.05 per 0.1 increase, 95% CI [1.04–1.07], *p* < 0.001) (Fig. 2c). We further adjusted for confounders, including PEEP and the P/F ratio, and the results demonstrated that the shape and nadir of the regression curve remained unchanged (Fig. 2d).

### The effect of VR on ICU mortality during different oxygenation states

**Fig. 3:**
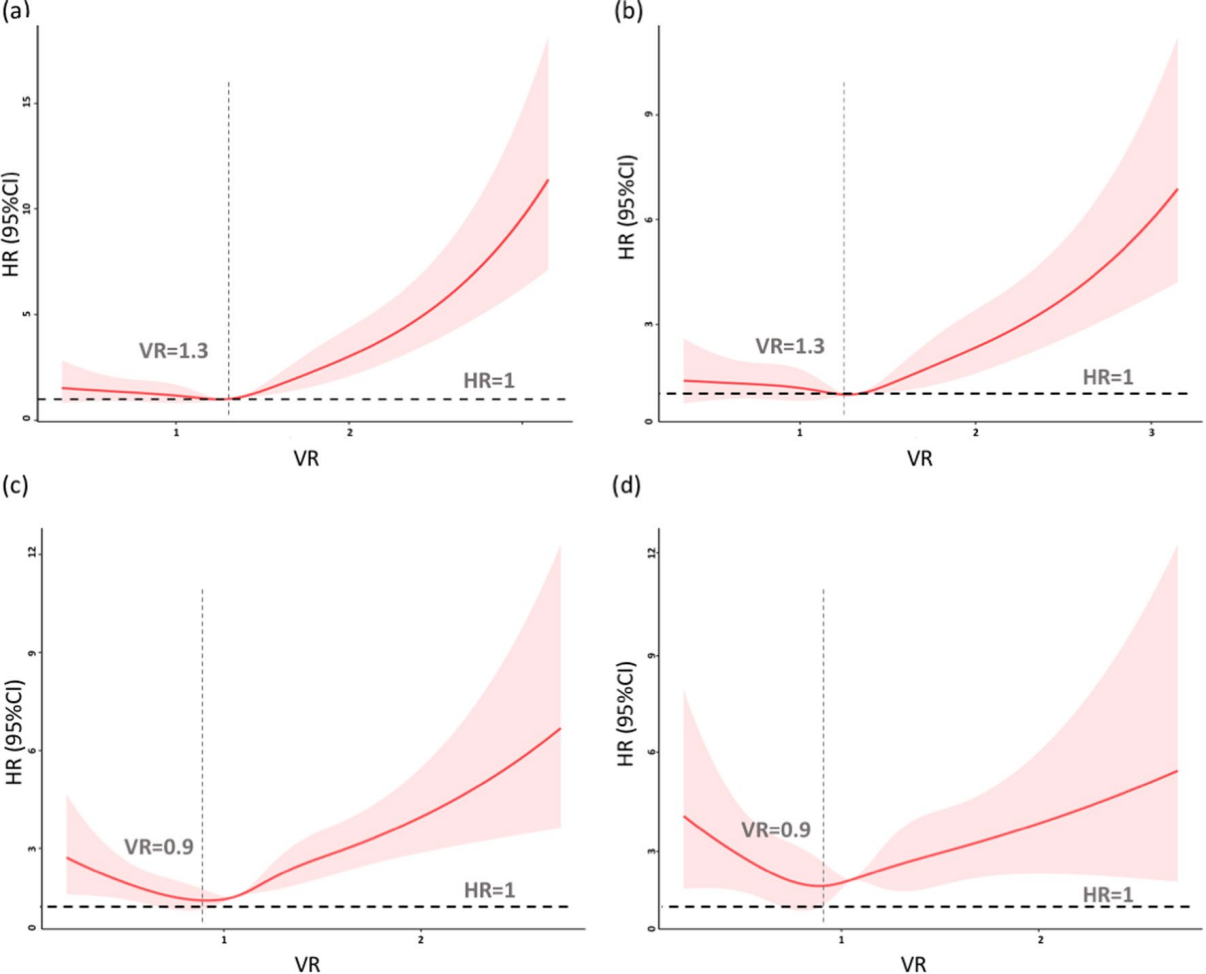
The nonlinear relationship of VR and the risk of 28-day mortality fit by univariate and multivariate Cox regression with RCS analyses. **a** Univariate Cox regression with RCS analyses showed that the risk of death remained unchanged until VR = 1.3 in patients with a P/F ratio < 200. When VR was greater than 1.3, the risk of death increased significantly with increasing VR (*p* < 0.001). **b** Multivariate Cox regression indicated that the nadir of the curve was 1.3 even though PEEP and the P/F ratio were adjusted in patients with a P/F ratio ≤ 200. **c** + **d** In patients with a P/F ratio ≥ 200, both univariate Cox regression (**c**) and multivariate Cox regression (**d**) indicated that the relationship between VR and the risk of death appeared as a J-shaped curve, with the lowest risk of mortality at VR = 0.9. When the VR was greater than 0.9, HR was positively correlated with VR, whereas negative correlations between VR and HR were identified when VR was less than 0.9. Shaded areas represent 95% confidence intervals

Since VR is commonly studied in patients with ARDS, we further investigated the effect of VR on ICU mortality during different oxygenation states. The patients were divided into a P/F ratio < 200 (severe hypoxemia group, SH group) and a P/F ratio ≥ 200 (nonsevere hypoxemia group, NSH group). In the SH group, the shapes of the fitting curves were similar to those obtained in the total ICU population, with the lowest risk for 28-day mortality occurring at the VR value of 1.3. When VR ≥ 1.3, there was an increase of 10% in HR for every 0.1 increase in VR (*p* = 0.000) and 7% in HR for every 0.1 increase in VR after adjustment for PEEP and the P/F ratio (*p* = 0.000) (Fig. 3a, b). In the NSH group, the relationship between VR and the risk of death presented a J pattern, with a nadir at 0.9. VR was positively related to 28-day mortality when VR was more than 0.9; however, VR correlated negatively with HR when VR was less than 0.9, and a 10% decrease in ICU mortality was observed for every 0.1 increase in VR (*p* = 0.034) (Fig. 3c). Multivariable adjusted models adjusted for PEEP and the P/F ratio revealed similar shapes of regression curves as those in univariate analysis (Fig. 3d).

### Subgroup analysis

**Fig. 4:**
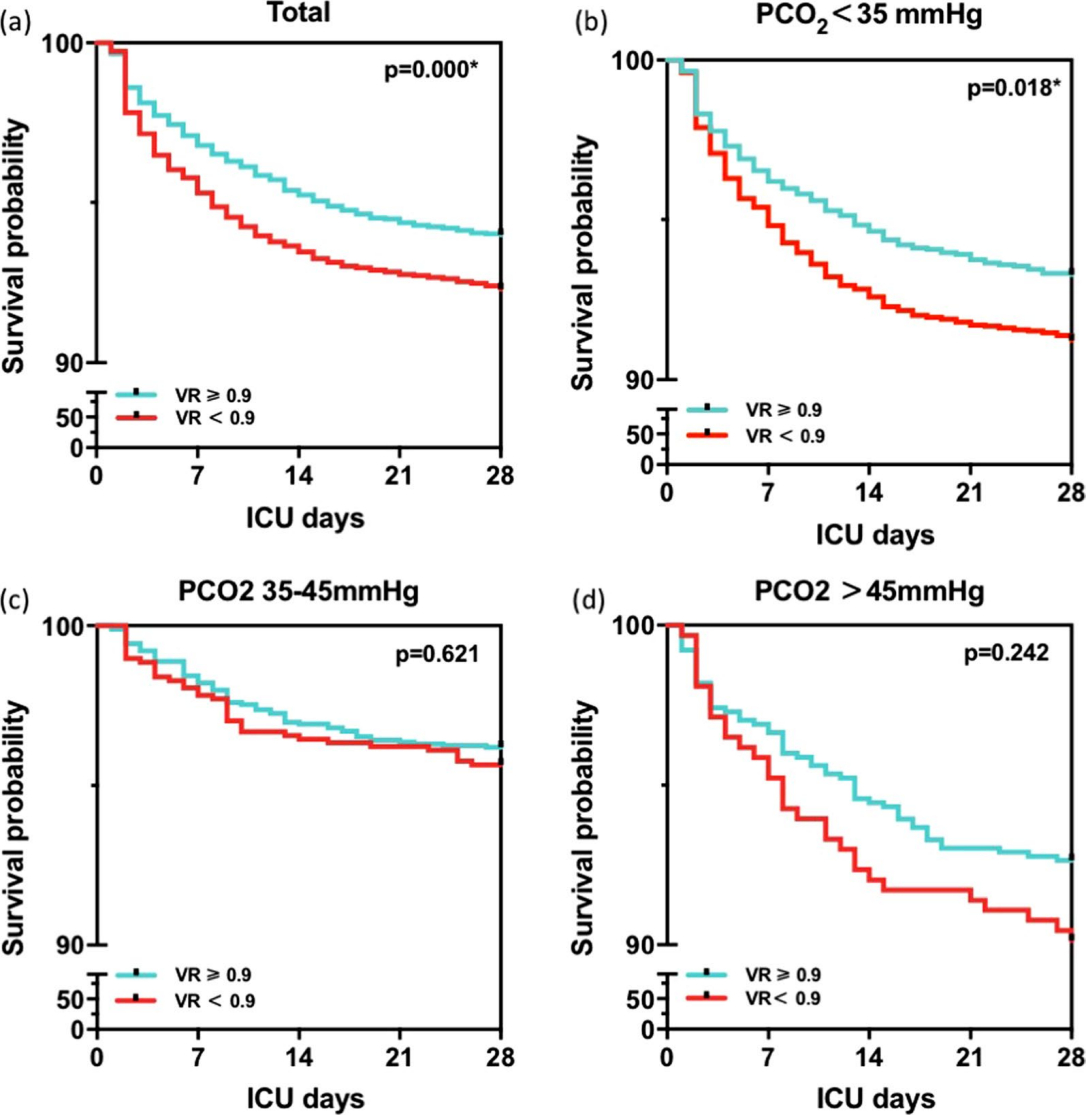
Survival analysis in different PCO_2_ groups of patients with a P/F ratio ≥ 200. In the population with a P/F ratio ≥ 200, survival analysis showed that the survival rate of the group with VR ≥ 0.9 was higher than that of VR < 0.9 (*p* = 0.000) (**a**). Supgroup analysis revealed a similar trend in patients with PCO_2_ < 35 mmHg as that in total population (*p* = 0.018) (**b**). However, no significant differences existed in the overall survival for the subgroup with PCO_2_ 35–45 mmHg (*p* = 0.493) (**c**) and PCO_2_ > 45 mmHg (*p* = 0.170) (**d**)

To further explain this phenomenon, we compared the respiratory parameters between survivors and nonsurvivors (Table 2). Table 2 shows that only PCO_2_ showed a significant difference (*p* = 0.014) between the two groups, while MV, RR, and PH were not significantly different. Finally, subgroup analysis was performed by dividing the NSH group into PCO_2_ < 35 mmHg, PCO_2_ 35–45 mmHg and PCO_2_ > 45 mmHg. Cox survival analysis adjusted for PEEP and P/F ratio showed that the survival probability of VR ≥ 0.9 was higher than VR < 0.9 in patients with P/F ratio ≥ 200, however, statistical significance was only achieved when PCO_2_ was less than 35 mmHg (*p* = 0.018, HR 1.382 [1.058, 1.804]) (Fig. 4).

**Table 2:** Respiratory parameters between survivors and nonsurvivors in patients with P/F ≥ 200 and VR < 0.9

Items	Survivor (N = 2107)	Non-survivor (N = 114)	*p* value
Age, mean(SD), years	57.8 (18.8)	59.0 (14.4)	0.957
Weight, median (IQR), kg	60.0 (54.0, 70.0)	64.0 (55.0, 71.0)	0.072
RR, median (IQR), bpm	13.7 (9.5, 15.1)	14.0 (8.9, 15.7)	0.099
Vte, median (IQR), ml	382.8 (349.3, 418.6)	373.1 (348.3, 416.1)	0.491
MV, median (IQR), l/min	5.0 (3.6,5.7)	5.1 (3.3, 5.9)	0.245
P/F Ratio, median (IQR), mmHg	402.9 (312.5, 492.0)	360.0 (284.2, 507.2)	0.145
PCO2, median (IQR), mmHg	34.4 (30.6,38.5)	33.0 (29.1, 37.9)	0.014
PH, median (IQR)	7.43 (7.38, 7.46)	7.42 (7.38,7.47)	0.733
VR, median (IQR)	0.76 (0.61, 0.84)	0.75 (0.54, 0.82)	0.001

## Discussion

The key results of this study are as follows: (1) VR was not only an important indicator of poor prognosis in SH patients but also an important independent prognostic predictor in an unselected ICU population. (2) The association between VR and 28-day mortality in the SH group was not consistent with that in the NSH group with regard to the shapes of the VR mortality curve and the cutoff point of VR for predicting mortality. (3) Among the ICU population with a P/F ratio greater than 200, VR was nonlinearly positively associated with 28-day mortality when VR ≥ 0.9 but inversely associated with the risk of death when VR was less than 0.9. A subgroup analysis showed increased mortality, with a lower VR only occurring in patients with hypocapnia. These findings may reflect the heterogeneity of VR for predicting ICU mortality across diverse populations. Moreover, the association between VR and mortality may be mostly influenced by the level of PCO_2_ in the ventilated population without severe hypoxemia.

Consistent with previous studies, our present study found that overall increased VR was associated with an increased risk of mortality, not only among patients with severe hypoxemia but also in general ICU patients. VR was proposed in recent years for bedside assessment of ventilatory efficiency, as it reflects the ability to clear CO_2_. According to the original formulas derived by Pratik Sinha et al., VR is jointly determined by Vd/Vt and CO_2_ production. Further study performed by the same team demonstrated physiological dead space, but not the production of CO_2_, was correlated with VR (modified correlation coefficients: 0.66 and 0.71). Thus, VR can indirectly reflect dead space. Increased dead space is common in ICU settings (e.g., ARDS, pulmonary embolism, COPD, and heart failure), especially in patients under mechanical ventilation [13]. Although dead space is not routinely measured in the ICU, it has long been recognized as a predictor of outcomes in patients with ARDS [14]. Recently, the ratio of the concentration of angiopoietin-2 relative to angiopoietin-1 has been identified as a useful vascular endothelial marker for predicting the severity of dead space fraction, indicating that pulmonary vascular endothelial damage and thrombus formation may be involved in the pathogenesis of decreased pulmonary blood flow and a subsequent increase in dead space [15].

VR was converted into a two-categorical variable using the median or mean values as the cutoff point in previous studies; however, it may not be capable of showing the relationship of VR and mortality since VR is a continuous variable and the trends of risk are nonlinear. Thus, we fit VR using restricted cubic splines with 5 knots to assess the dose–response relationship between VR and the risk of death. Compared to the population with SH, the VR corresponding to the minimum level of mortality was lower in the NSH group, suggesting ventilation heterogeneity between these two groups. Interestingly, in populations with a P/F ratio ≥ 200, our study found that there was a paradoxical inverse relationship between VR and overall mortality at VR of less than 0.9, contradicting the predictions of higher mortality with increased VR in previous studies. Further subgroup analysis found that this phenomenon, which looked like “pseudonormalization of a VR”, occurred only when PCO_2_ < 35 mmHg (hypocapnia).

Previous studies also found that hypocapnia was associated with higher ICU mortality in patients with pneumonia and ARDS [16–18]. Intriguingly, a similar result was observed in one of the recent studies by Fabiana Madotto et al., which showed that hypocapnia was common in ventilated ARDS patients (12.7%), and higher ICU mortality was observed in mild to moderate ARDS patients with PaCO_2_ < 35 mmHg during the first 48 h compared with normocapnia [19]. Several possible explanations might account for an inverse association between VR and ICU mortality in the NSH group under hypocapnia. First, according to previous studies, a lower VR should represent a better ability of the lung to clear CO_2_ if VR could reflect VD/VT. We hypothesize that the better CO_2_ clearance in the population without hypoxemia may be attributed to a compensatory increase in respiratory drive or hyperventilation under excessive mechanical ventilation support to obtain normal oxygenation. The role of respiratory drive is being increasingly recognized as an independent predictor of lung injury in critically ill patients due to the weakening of the respiratory muscles [20, 21]. Thus, a relatively normal VR or P/F ratio might not accurately reflect the real clinical state under this situation. Second, despite the positive relationship between VR and VD/VT with a significant difference, the correlation coefficient was not very high (r = 0.66), and no study has focused on the relationship between VR and VD/VT in different PCO_2_ categories in an ICU population [10]. Therefore, VR may be unable to reflect the dead space in this case. These results alert us to use VR to replace VD/VT with caution under specific clinical scenarios. Finally, we summarized a proof-of-concept classification of VR in population on mechanical ventilation (shown in Table 3) according to P/F ratio.

**Table 3:** A proof-of-concept classification of VR on the mortality according to the P/F ratio in a mechanically ventilated population

Group	P/F ratio ≥ 200 mmHg	P/F ratio < 200 mmHg
Relationship of VR and ICU mortality	J-shaped dose–response association	Positive correlation
Cutoff of VR	0.9	1.3
Pathophysiology meaning of VR	When VR < 0.9, a decreased VR may indicate hyperventilation under excessive mechanical ventilation support. Moreover, the value of VR was impacted by hypocapnia. Hence, a low VR might be related to a poor outcome When VR > 0.9, the VR was positively related to mortality	An increase in VR might reflect an increased dead space level. Hence, a high VR indicates severe lung injury and a poor outcome

Our study has several limitations. First, this study was a single-center retrospective study, which may not be representative of the overall sample and the level of evidence may not be sufficient to obtain the causal relationship between the two variables, therefore, a multicenter, prospective study should be planned in the future. Second, VR has been studied mainly in the ARDS population in previous studies, but unfortunately, the precise number of ARDS patients was unavailable in the present study. Further study is required to validate the described cutoff value in the ARDS patients. Third, the patients we included in our study were all undergoing mechanical ventilation, and we did not group these mechanical ventilation patients according to the mechanical ventilation pattern, e.g., controlling ventilation mode and autonomous breathing mode, so we could not see the effect of mechanical ventilation mode on VR. Fourth, we did not calculate the physiologic dead space simultaneously by measuring PĒCO_2_ in the population without severe hypoxemia to determine whether VR can also reflect physiologically dead cavities in nonhypoxic populations. Fifth, because this is a retrospective study, all variables were not collected at the same time, which may result in bias in data analysis. With the aim to reduce the variation of VR with time, the average values of each variable were calculated based on the data collected 12–18 h after the onset of mechanical ventilation.

## Conclusions

VR is a good predictor of mortality risk in patients with severe hypoxemia as well as in an unselected ICU population, but the shapes of the VR mortality curve varied by oxygenation status. Among ICU populations without severe hypoxemia, both VR values that were too high or too low were associated with increased mortality. Pursuing a low VR may result in better outcomes in the severe hypoxemia condition, and a mild value of VR might be beneficial in the non-severe hypoxemia condition, further prospective and interventional studies are required.

## Data Availability

All the other data supporting the findings of this study are available within the article and from the corresponding author upon reasonable request.

## Abbreviations

VR: Ventilatory ratio
ARDS: Acute respiratory distress syndrome
ICU: Intensive care unit
RCS: Restricted cubic spline
VD/VT: The physiological dead-space fraction
PBW: Predicted body weight
APACHE II: Acute Physiology and Chronic Health Evaluation score II
ScvO2: Central venous oxygen saturation
CVP: Central venous pressure
Lac: Lactate
PI: Perfusion index
RR: Respiratory rate
Vte: Expiratory tidal volume
Vte: Inspiratory tidal volume
Ppeak: Peak inspiratory airway pressure
Pmean: Mean airway pressure
P/F Ratio: PaO2 (partial pressure of oxygen)/FiO2 (the fraction of inspired oxygen) ratio
APACHE: Acute Physiology and Chronic Health Evaluation
SOFA: Sequential Organ Failure Assessment
HR: Hazard ratio
KM: Kaplan–Meier
Cox regression: Cox proportional hazards regression
